# Primary aldosteronism patients show skin alterations and abnormal activation of glucocorticoid receptor in keratinocytes

**DOI:** 10.1038/s41598-017-16216-5

**Published:** 2017-11-17

**Authors:** Julia Boix, Judit Bigas, Lisa M. Sevilla, Maurizio Iacobone, Marilisa Citton, Francesca Torresan, Brasilina Caroccia, Gian Paolo Rossi, Paloma Pérez

**Affiliations:** 1 Instituto de Biomedicina de Valencia-Consejo Superior de Investigaciones Científicas (IBV-CSIC), Jaime Roig 11, E-46010 Valencia, Spain; 2 Endocrine Surgery Dept. of Surgical and Oncological Sciences, Dept. of Medicine, University of Padua, Padova, Italy; 3 Clinica dell’Ipertensione Arteriosa, Dept. of Medicine -DIMED, University of Padua, Padova, Italy

## Abstract

Primary aldosteronism (PA) is a disease characterized by high aldosterone levels caused by benign adrenal tumors being the most frequent cause of secondary hypertension. Aldosterone plays vital physiological roles through the mineralocorticoid receptor (MR) but in certain cell types, it can also activate the glucocorticoid (GC) receptor (GR). Both MR and GR are structurally and functionally related and belong to the same family of ligand-dependent transcription factors that recognize identical GC regulatory elements (GREs) on their target genes. GCs play key roles in skin pathophysiology acting through both GR and MR; however, the effects of aldosterone and the potential association of PA and skin disease were not previously addressed. Skin samples from PA revealed histopathological alterations relative to control subjects, featuring epidermal hyperplasia, impaired differentiation, and increased dermal infiltrates, correlating with increased NF-κB signaling and up-regulation of TNF-A and IL-6 cytokines. PA skin samples also showed significantly higher expression of MR, GR, and HSD11B2. In cultured keratinocytes, aldosterone treatment increased GRE transcriptional activity which was significantly inhibited by co-treatment with GR- and MR-antagonists. This study demonstrates that high levels of aldosterone in PA patients correlate with skin anomalies and inflammatory features associated with abnormal GR/MR activation in epidermal keratinocytes.

## Introduction

Primary aldosteronism (PA) is characterized by high aldosterone plasma levels and low plasma renin and is now recognized as one of the more common causes of secondary hypertension (5–15% of all hypertensive patients)^1^. The underlying cause in many PA patients is unilateral aldosterone-producing adenoma (referred to as Conn’s adenoma), although it can also be caused by bilateral and unilateral adrenal hyperplasia. The morbidity and mortality associated with PA primarily, besides hypokalemia and hypertension, are related to the fact that for the same degree of blood-pressure elevation PA patients develop more prominent cardiovascular and renal damage, as compared to matched patients with essential hypertension^2^. Nonetheless, patients with unilateral causes of PA can be long-term cured and have their cardiovascular damage regressed by laparoscopic adrenalectomy, while those with bilateral disease require life-long treatment with mineralocorticoid receptor (MR) antagonists^3^.

Aldosterone plays its main physiological role by retaining sodium and water, and excreting potassium in the kidney via MR, the ancestor hormone receptor from which the other steroid receptors, including the glucocorticoid (GC) receptor (GR), have evolved^4,5^. Both MR and GR belong to the nuclear receptor subfamily NR3C and act as ligand-dependent transcription factors, which modulate transcription on target genes by binding to identical GC regulatory elements (GREs)^6–8^. MR also has high affinity for binding cortisol, which circulates at 100–1000-fold higher concentration than aldosterone; however, in aldosterone target tissues this is prevented by the action of the microsomal enzyme 11β-hydroxysteroid dehydrogenase (HSD11B2), which converts GCs into their inactive metabolites^5^. Adding further complexity to this scenario, aldosterone at supra-physiological concentrations can also bind and activate GR^9^.

Finally, both aldosterone and GCs can also exert rapid, –also known as non-genomic–actions that cannot be explained by the classic genomic effects through the classic nuclear hormone receptors. However, it is still debated whether these rapid effects are mediated through membrane GR or MR, other unknown membrane receptors, or in the case of the rapid effects of aldosterone in the cardiovascular system, the G-protein coupled receptor GPER-1^10^.

It is widely accepted that MR over-activation is involved in inflammation, fibrosis, oxidative stress, and aging in renal and cardiovascular tissues. In fact, the use of pharmacological MR antagonists has proven effective in reducing the morbidity and mortality of patients with cardiovascular disease in several clinical trials^11^. In the case of incomplete remission PA patients are also treated with MR antagonists^5^.

While GR function in the skin has been extensively documented^12–16^, that of the MR has only recently received some attention^17–22^. Overall, data from genetically modified mouse models and human skin biopsies demonstrated skin atrophy in response to inappropriate activation of both MR and GR by high doses of GCs^18–21^. Moreover, aldosterone can favor collagen and elastin deposition in human skin via MR-dependent and –independent actions, respectively, which in the case of elastin can be reversed by MR antagonists resulting in improved dermal remodeling^23^. However, to the best of our knowledge, a direct correlation between PA and altered skin conditions has not been previously investigated.

We have therefore investigated the skin architecture and function of PA patients relative to that of control individuals with non-secreting adrenocortical tumors. Our studies include histopathological analysis as well as quantitative (q) RT-PCR assessment of inflammatory markers *in vivo*. Additionally, we performed experiments in cultured keratinocytes treated with aldosterone to evaluate whether GR activation in hyperaldosteronism occurred specifically in the epidermal compartment.

## Results

### Primary aldosteronism (PA) patients show skin alterations and inflammatory features

**Figure 1:**
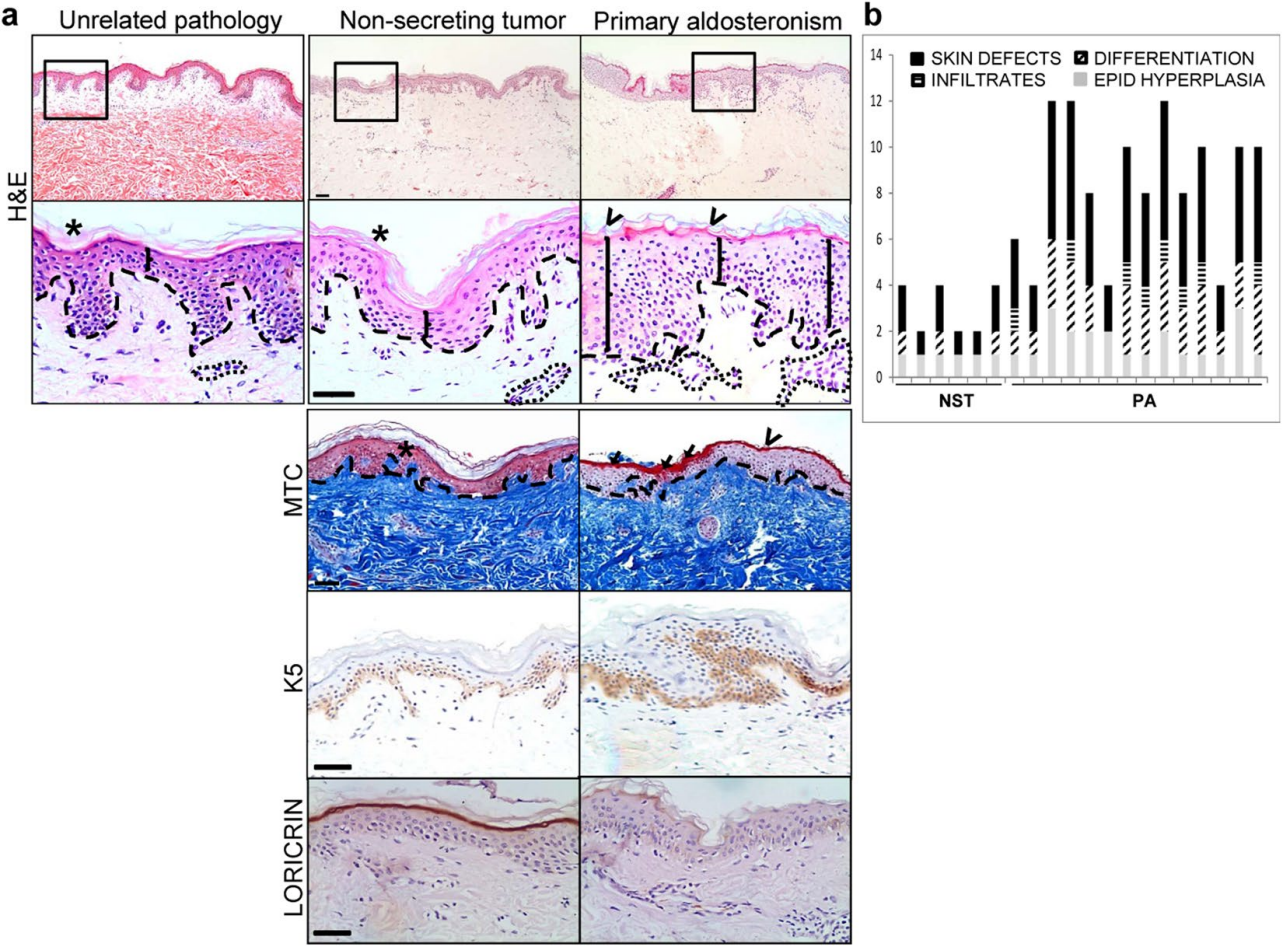
Altered skin histopathology in primary aldosteronism patients. (**a**) Skin samples from individuals with an unrelated pathology (left), bearing non-secreting adrenocortical tumors (middle, n = 6) or aldosterone-producing ademomas (right, n = 14) were assessed by hematoxilin/eosin (H&E), Masson trichromic staining (MTC), or by immunostaining with specific antibodies. Keratin (K)5 is normally expressed in epidermal proliferating (basal) keratinocytes while loricrin expression is restricted to differentiated (suprabasal) keratinocytes. The discontinuous line delimitates epidermis and dermis. Bracket, epidermal width; *normal stratum corneum; open arrow, abnormal stratum corneum; arrow, parakeratosis; dotted line, dermal infiltrate. Bar: 50 μm. (**b**) Graphical representation of histopathological assessment of skin alterations in individuals with non-secreting tumors (NST) and primary aldosteronism (PA) in Table 1. Data include the score of epidermal hyperplasia, degree of differentiation, presence of infiltrates, and overall skin defects (as the sum of partial scores).

To investigate the *in vivo* impact of increased circulating aldosterone on human skin homeostasis, at the time of unilateral laparoscopic adrenalectomy we collected skin specimens from consecutive patients with PA (n = 14) caused by an aldosterone producing adenoma and from patients with non-secreting adrenocortical tumors (n = 6), used as controls. The histopathological assessment of skin samples scored epidermal hyperplasia, degree of epidermal differentiation, and the presence of immune infiltrates (Table 1 and Fig. 1a,b).

**Table 1:** Patients’ data and histopathological assessment of skin alterations.

DIAGNOSIS	GENDER	AGE	High BP	^1^EPID HYP	^2^DIFFERENTIATION	^3^INFILTRATES	SKIN DEFECTS
NST	F	65	Y	1	0	0	1
NST	M	59	N	1	0	0	1
NST	F	71	Y	1	0	0	1
NST	M	63	N	1	1	0	2
NST	F	54	Y	1	1	0	2
NST	M	84	Y	1	1	0	2
PA	F	77	Y	1	1	0	2
PA	M	62	Y	2	0	0	2
PA	M	52	Y	1	1	0	2
PA	M	64	Y	1	1	1	3
PA	M	45	Y	2	2	0	4
PA	M	48	Y	1	2	1	4
PA	M	71	Y	1	2	1	4
PA	F	67	Y	1	3	1	5
PA	M	52	Y	1	3	1	5
PA	F	56	Y	3	2	0	5
PA	F	54	Y	1	3	1	5
PA	M	73	Y	2	3	1	6
PA	M	55	Y	3	3	0	6
PA	M	49	Y	2	3	1	6

Notes: High blood pressure (BP): Y, yes; N: no. ^1^Score for epidermal hyperplasia. Normal: 1; Focal hyperplasia: 2; Extended hyperplasia (>30%): 3. ^2^Score for degree of differentiation (DIFF). Well differentiated: 0; Focal alterations: 1–2; Poorly differentiated: 3. ^3^Infiltrates. Yes: 1; No: 2. Overall skin defects are the sum of partial scores 1–3. Normal: 0–3. Impaired: >4.

Abbreviations: PA: primary aldosteronism; NST: non-secreting tumor.

Hematoxylin/eosin (H&E) staining of skin samples revealed that individuals with non-secreting tumors had normal tissue architecture featuring relatively well differentiated epidermis consisting in 4–5 cell layers with compact stratum corneum and little cellularity in the dermal compartment^24^ (Fig. 1a, middle panel, H&E). Consistently, the expression of keratin (K)5 was restricted to the epidermal basal layer, the only layer in which keratinocytes actively proliferate, while loricrin, a marker of epidermal differentiation, was localized in the more suprabasal layers (Fig. 1a, middle panel, K5, loricrin). As an additional control, we used human skin samples obtained from subjects undergoing surgery for an unrelated pathology, which confirmed that the skin of individuals bearing non-secreting tumors have normal histological features (Fig. 1a, left panel, H&E).

At variance, the skin of PA patients showed alterations that included areas with increased epidermal width, impaired differentiation, and frequent abnormally high dermal infiltrates (Fig. 1a, right panel, H&E, and 1b). Epidermal hyperplasia was coincident with abnormal expression of K5 in the first suprabasal layers and also with poor epidermal differentiation correlating with the focal loss of loricrin (Fig. 1, right panel, K5, loricrin). In a subset (approximately 15%) of PA patients impaired differentiation also included areas with parakeratosis (retention of nuclei in the SC), which, along with epidermal hyperplasia and high immune infiltrates, are hallmarks of psoriasis^25^.

Despite excess circulating aldosterone in PA patients, Masson trichromic staining showed no major differences in dermal collagen deposition relative to control subjects suggesting that high aldosterone mainly affects epidermal homeostasis (Fig. 1, MTC).

To assess possible differences in epidermal pigmentation of PA relative to control skin, we performed Fontana-Masson staining and found an overall increase of melanin patches in the skin of PA patients, as shown by representative images and scoring of the skin samples (Fig. S1).

**Figure 2:**
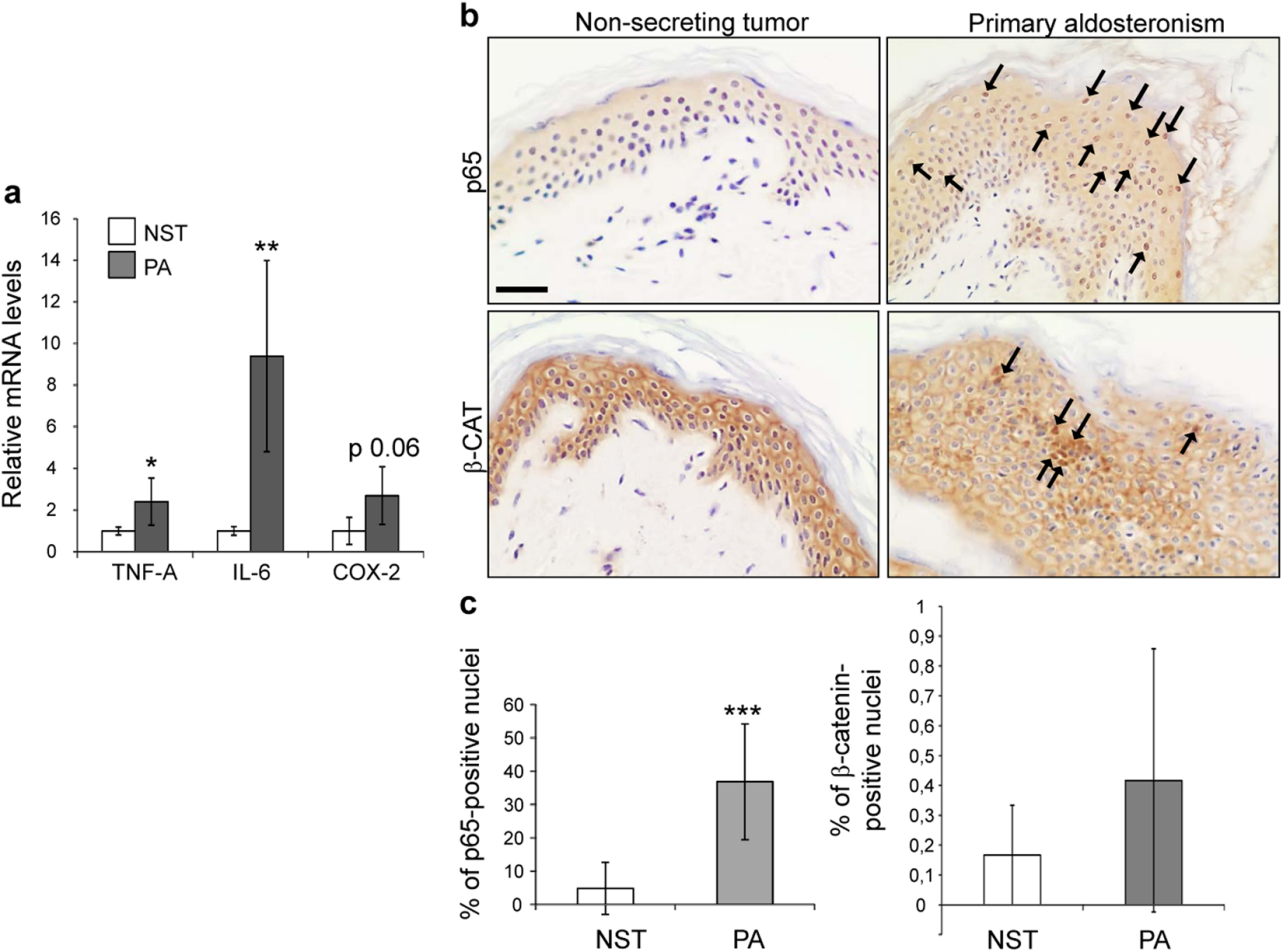
Up-regulation of pro-inflammatory cytokines and increased NF-κB signaling in the skin of primary aldosteronism patients. (**a**) Relative mRNA levels of *TNF-A*, *IL-6*, and *COX-2* were assessed in the skin of primary aldosteronism patients and control subjects. Mean values ± SD are shown; n ≥ 3 per group. Student’s *t* test; *p < 0.05; **p < 0.01. (**b**) Immunostaining using p65-NF-κB or β-catenin specific antibodies showed increased nuclear localization of both proteins in the skin samples of PA patients relative to controls. Bar: 50 μm. (**c**) Quantitation of positive p65-NF-κB or β-catenin nuclei in the epidermis of PA patients and control skin sections. Mean values ± SD are shown; n > 3 per group. Student’s *t* test; ***p < 0.001.

As the relatively high dermal immune infiltrates in the skin of PA patients suggested chronic inflammation, we assessed the mRNA levels of the pro-inflammatory cytokines *TNF-A* and *IL-6*. This analysis showed that they were up-regulated by 2.5- and 9-fold, respectively, relative to control subjects (Fig. 2a). We also found that the mRNA levels of *COX-2*, a key enzyme in prostaglandin biosynthesis, were more than 2-fold higher in PA patients’ skin, albeit without reaching statistical significance (Fig. 2a). Since most inflammatory mediators are transcriptionally induced by the transcription factor NF-κB^26^, we examined the expression of p65-NF-κB, the family member with higher transactivation capabilities. Immunostaining showed increased nuclear localization of p65 both in the epidermal layers and the dermal compartment of PA patients´ skin relative to controls (Fig. 2b; p65, arrows). In epidermal keratinocytes, this increase was statistically significant as shown by 5- to 6-fold higher number of positive p65 nuclei in PA relative to controls (Fig. 2c; p65).

It is known that up to 70% of aldosterone-producing adenomas present with constitutive WNT/β-catenin activation^27^, however, whether this signaling pathway was altered in patients´ skin was unknown. We detected β-catenin localization at the membrane of suprabasal keratinocytes and in the cytoplasm of basal keratinocytes of both PA and controls by immunostaining (Fig. 2b; β-catenin). However, in the skin of PA patients we also detected nuclear β-catenin in suprabasal keratinocytes, suggesting that it may contribute to transcriptional activation of WNT-responsive genes. The nuclear localization of β-catenin in the epidermis of PA individuals was not a generalized feature but was rather found in discrete sections of patients’ skin samples. Therefore, upon quantification of β-catenin-positive nuclei, the slight increase observed in PA keratinocytes relative to those of controls did not reach statistical significance (Fig. 2b,c; β-catenin).

### Increased GR expression and activity in skin samples from PA patients

**Figure 3:**
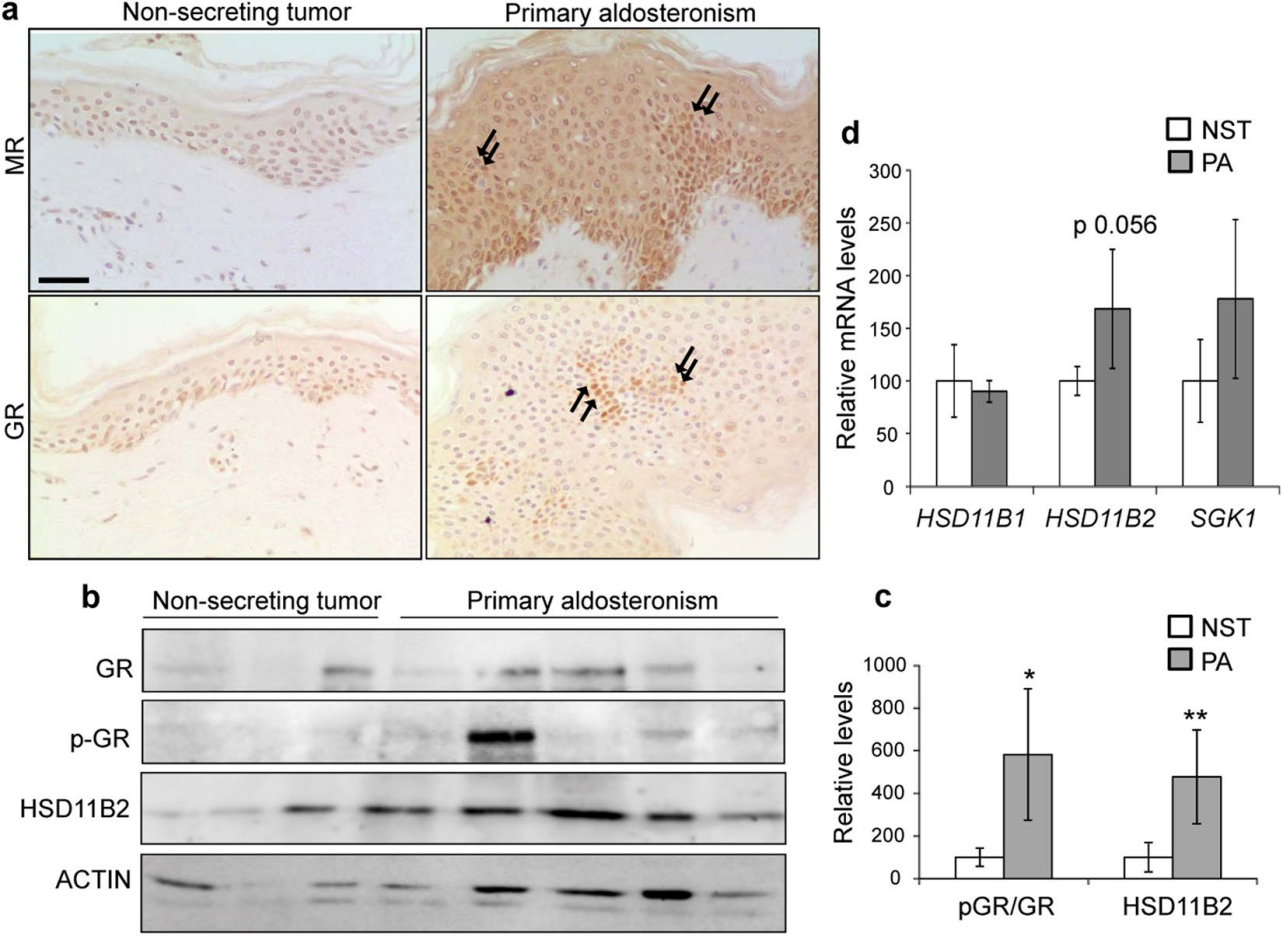
Increased GR expression and activity in skin samples from primary aldosteronism patients. (**a**) Immunostaining demonstrated focal increased nuclear MR and GR staining in skin samples from PA patients relative to control subjects. Bar: 50 μm. (**b**) Immunoblotting using specific antibodies and (**c**) quantitation showed augmented overall levels of GR, activated GR (p-GR), and HSD11B2 expression in skin samples from PA patients. Mean values ± SD are shown; n ≥ 3 per group. Student’s *t* test; *p < 0.05; **p < 0.01. (**d**) Relative mRNA levels of *HSD11B1*, *HSD11B2*, and *SGK1* were assessed in the skin of PA patients and control subjects. Mean values ± SD are shown; n > 3 per group. Student’s *t* test.

We next assessed the expression of both MR and GR by immunostaining and found focal increased nuclear localization of both transcription factors in skin samples from PA patients, coinciding with areas of epidermal hyperplasia (Fig. 3a). MR activation was likely due to the excess of circulating aldosterone in PA patients and may contribute to the observed skin inflammation (Fig. 2). However, since the increased nuclear GR staining in epidermal keratinocytes was unexpected, we wondered if it also correlated with increased GR activity and, therefore, examined total (GR) and phosphorylated (p-GR) proteins. The p-GR/GR ratio was significantly increased (3- to 5-fold) in 75% of PA patients’ skin samples as quantitated by Western blot (Fig. 3b,c).

We also examined HSD11B2 expression and found relatively low levels in the skin of control subjects (Fig. 3b). This finding is in agreement with previous data in human epidermis^28^. In contrast, HSD11B2 expression was up-regulated 3- to 4-fold in 78% of skin samples from PA patients (Fig. 3b,c), suggesting an effect of long-term exposure to high aldosterone on the regulation of this enzyme.

Besides HSD11B2, GC availability is modulated by 11β-hydroxysteroid dehydrogenase 1 (HSD11B1), a bidirectional enzyme that interconverts active into inactive GCs, which *in vivo* predominantly generates active GCs. Thus, imbalances in the relative expression/activity of HSD11B1 and HSD11B2 have an impact in the GR- and MR-mediated transcriptional responses, affecting skin homeostasis and inflammation. We assessed the expression of *HSD11B1* transcript by RT-qPCR and found no differences between control and PA skin (Fig. 3d). On the other hand, *HSD11B2* mRNA levels showed an increased expression in PA patients that was nearly statistically significant (p = 0.056; Fig. 3d).

We reasoned that the relative increase of HSD11B2 expression in the skin of PA patients may lead to reduced effects of GCs and increased effects of aldosterone via MR and GR ultimately contributing to the pathological abnormalities observed in this tissue. To evaluate this, we assessed the expression of the serum- and glucocorticoid-inducible kinase (*SGK1*), a common transcriptional target of both receptors, by RT-qPCR. Our data show that although there was a trend towards an increase of the *SGK1* mRNA levels in PA patients relative to controls, the differences were not statistically significant (Fig. 3d).

### GR is required for Aldosterone-induced GRE-dependent transcriptional response

To test whether GR plays specific roles in the epidermal compartment in response to excess aldosterone, we cultured epidermal keratinocytes and examined hormone-induced nuclear translocation by cell fractionation and immunoblotting, as well as by immunofluorescence. While the use of the keratinocyte cell line allows for mechanistic studies using pharmacological inhibitors, it does not mimic the pathophysiology in the skin of PA patients.

**Figure 4:**
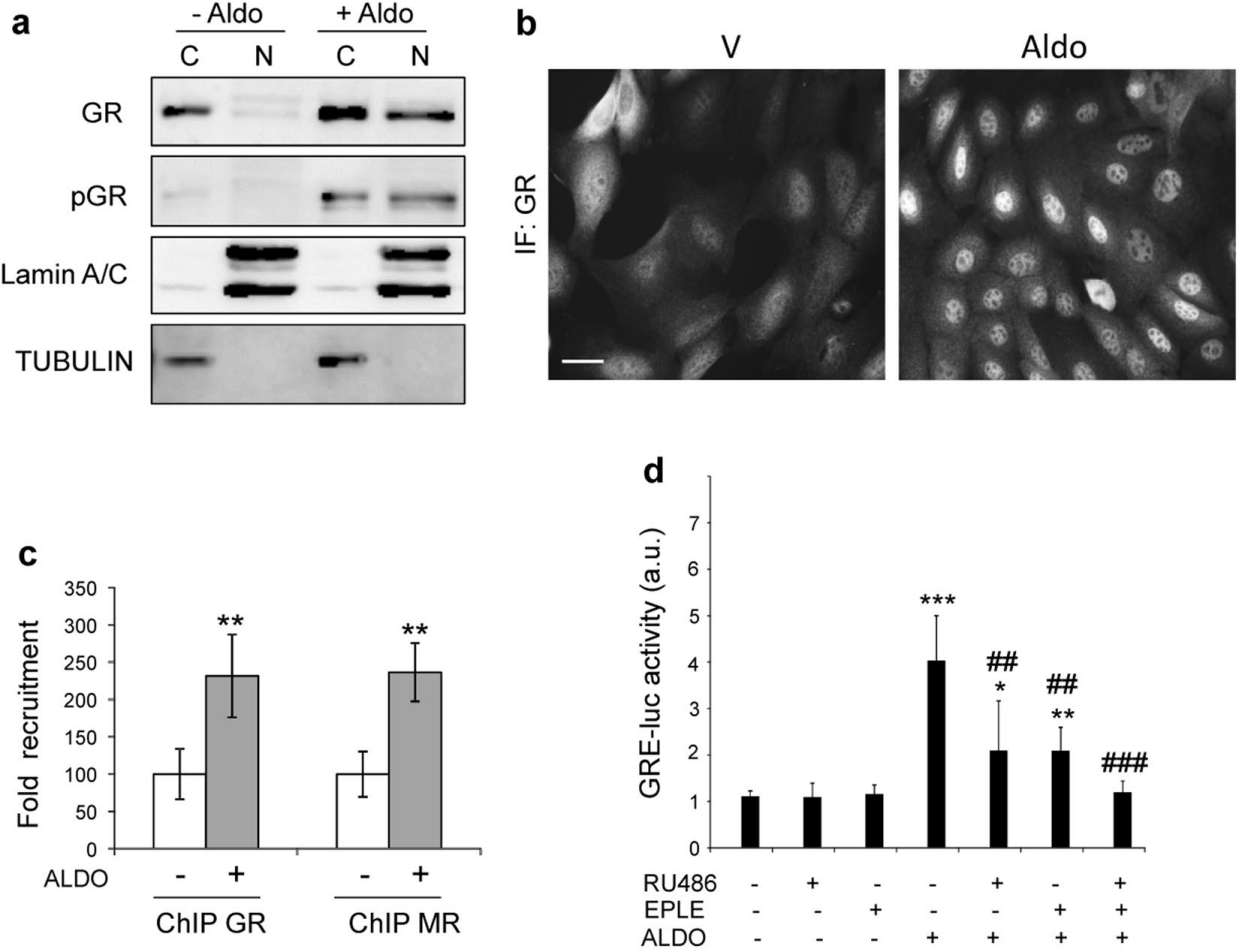
GR is required for aldosterone-induced GRE-dependent transcriptional response in cultured keratinocytes. Cultured keratinocytes were treated with vehicle or aldosterone as indicated. (**a**) Expression of total and phosphorylated (p-) GR of cytoplasmic (C) and nuclear (N) fractions was assessed by Western blot in the absence or presence of aldosterone (100 nM, 1 h). Lamin A/C and Tubulin are shown as control of cell fractionation. (**b**) GR subcellular localization was assessed by immunofluorescence in the absence or presence of aldosterone (100 nM, 1 h). (**c**) ChIP qPCR assessing GR and MR recruitment to GRE-containing *Gilz* regulatory sequences in vehicle- or aldosterone-treated keratinocytes (3 nM, 2 h). Mean values ± SD are shown; n = 3 per treatment. Student’s *t* test; **p < 0.01. (**d**) GRE-mediated transcriptional activity was evaluated after transfecting keratinocytes with GRE-luciferase reporter in the presence of vehicle or aldosterone (1 μM, 6 h) and the indicated antagonists (RU486, 10 μM, 16 h), eplerenone (10 μM, 16 h) or both (1 μM each, 16 h). Mean values ± SD are shown; n = 3 per treatment. Post hoc Tukey test; *p < 0.05; **, ^##^p < 0.01; ***, ^###^p < 0.001. Asterisks indicate significant differences relative to untreated group and hashes denote significant differences relative to aldosterone-treated cells.

Our results indicate that upon aldosterone incubation, GR was phosphorylated and translocated to the nucleus (Fig. 4a,b). To confirm that aldosterone-induced nuclear GR is functional, we first assessed its recruitment to GRE-containing regulatory sequences of *Gilz* (a target of both GR and MR) by chromatin-immunoprecipitation followed by QPCR using specific primers (Fig. 4c). Our data showed 2.2-fold increase in GR recruitment in the presence of aldosterone relative to vehicle; the recruitment of MR (2.3-fold) after aldosterone treatment was very similar to that of GR (Fig. 4c).

We checked whether aldosterone-triggered activation of GR was capable of inducing GRE-mediated transcriptional activity in cultured keratinocytes. After transfection with a GRE-luciferase reporter cells were incubated in the presence of vehicle, aldosterone, the GR antagonist RU486, or the MR antagonist eplerenone. While aldosterone significantly induced GRE-luciferase activity (4-fold), the antagonists alone had no effect (Fig. 4d). However, the combined treatment with aldosterone plus RU486 (10 μM) or aldosterone plus eplerenone (10 μM) significantly decreased the GRE-luciferase activity (Fig. 4d). Also, treatments with aldosterone plus RU486 plus eplerenone (1 μM each) caused more decrease in the GRE-luciferase activity than separately, such that there was effectively no induction relative to the aldosterone-treated samples (Fig. 4d). Altogether, these results in cultured keratinocytes support the above findings in PA patients in that aldosterone overproduction activates GR in epidermal keratinocytes and contributes to alterations in epidermal homeostasis.

## Discussion

Steroid hormones play key roles in skin homeostasis and inflammatory conditions^29^. Thus, understanding the relative roles of GR and MR, two closely related hormone receptors, in this tissue is increasingly becoming a focus of attention^14,19,20^. Although we have investigated the transcriptional function of GR and MR upon aldosterone treatment, we cannot rule out that some of the reported findings are due to a rapid pathway mediated through membrane GR or MR, or other unknown membrane receptors^10^.

A link between increased levels of plasma aldosterone concentration and psoriasis and atopic dermatitis has been established more than three decades ago^30^. Moreover, a correlation between hyperaldosteronism and severe atopic dermatitis has been reported in infants with pseudohypoaldosteronism type I (PHA-I), in which electrolyte abnormalities and hypovolemia led to dehydration, hypoproteinemia and hyponatremia^31^. Overall, available findings suggest that aldosterone can play a permissive role in the onset and/or progression of prevalent skin inflammatory diseases.

Notwithstanding this, the impact of increased plasma aldosterone levels on the skin of PA patients was not previously addressed to the best of our knowledge. Histopathological examination of skin specimens from adrenalectomized patients demonstrated that 71% of the PA patients (Table 1, and Figs 1 and 2) showed skin alterations, which included increased epidermal width, impaired differentiation, and frequent abnormally high dermal infiltrates. In approximately 15% of the PA patients analyzed, skin defects were more pronounced, with areas of parakeratosis, which together with increased epidermal width and immune infiltrates resembled psoriasis-like features (Table 1 and Fig. 1). Regarding the epidermal hyperpigmentation observed in PA patients (Fig. S1), one might speculate that it is related to post-inflammatory events, as this is a common late sequela in several inflammatory skin conditions such as eczema and psoriasis. However, additional information regarding the UV exposition of all individuals analyzed would be required to reach definitive conclusions.

These PA patients also showed signs of chronic inflammation and oxidative stress, including increased NF-κB signaling and significant up-regulation of mRNA levels of the pro-inflammatory cytokines *TNF-A* and *IL-6* in the skin (Fig. 2), in keeping with that reported in hyperaldosteronism in other tissues, where high aldosterone levels exert pro-inflammatory effects^11^. Hence, hyperaldosteronism might induce MR nuclear localization in the skin and contribute to the observed skin alterations and inflammation (Fig. 2).

As GCs can activate both MR and GR and the latter can be activated also by aldosterone in certain cell types^32,33^ (Fig. 4), and considering that *in vivo* even in PA patients the plasma levels of aldosterone are much lower than those of cortisol, it might be argued that it is unlikely that in the skin the GR is occupied by aldosterone. However, in some cell types this has been shown to occur upon stress signalling mediated by tissue-specificity of transcription factors and/or co-regulators. For instance, in the distal nephron, and within the range of physiological aldosterone concentrations, sodium transport is controlled by MR and GR occupancy during salt restriction or acute stress^33^. Furthermore, it was shown that aldosterone induced nuclear localization of GR and up-regulated connective tissue growth factor (*CTGF)*, a known aldosterone-target in cultured mesangial cells, two effects that were specifically inhibited by GR but not MR antagonists^32^. In addition, it was also reported that aldosterone induced clonal β-cell failure through the GR^33^. The present findings in epidermal keratinocytes show that in response to aldosterone, both GR and MR contribute to GRE transactivation, thus implying that transcriptional regulation can occur through MR, GR, or both, in cell-type specific manners (Fig. 4).

The increased trend in SGK1 up-regulation in the skin of PA patients is consistent with an overall augmented transcriptional activity of both GR and MR in the context of high aldosterone levels. Our results are also compatible with the pro-inflammatory role of aldosterone in several tissues being mediated by SGK1-induced NF-κB activity, which leads to increased expression of inflammatory mediators^11^.

The finding that HSD11B2 expression was up-regulated in the majority of PA patients’ skin samples relative to controls (Fig. 3b) is intriguing in that while low expression of this cortisol-inactivating enzyme allows for MR occupancy by GCs, theoretically increased levels of HSD11B2 could favor the binding of aldosterone to the MR. Hence, enhanced HSD11B2 expression and activity can result in increased aldosterone availability for MR in skin thus exposing this tissue to the pro-inflammatory and pro-fibrotic effects of the hormone.

## Materials and Methods

### Human samples

The study was performed in 20 patients appointed for surgery (adrenalectomy) at the Hospital of Padova. Clinical data and histopathological skin assessment of patients are summarized in Table 1. Abdominal skin specimens were collected at the time of surgery and fixed in 70% ethanol for histopathological analysis or immediately frozen at −80 °C until RNA or protein extraction. The Study was approved by the Ethics Committee of the University of Padova and patients signed a written informed consent to participate in the study. Laparoscopic adrenalectomies were always performed in the morning between 8 and 11, which rules out differences due to skin oscillatory rhythms.

### Histological analysis

Fixed skin specimens were paraffin-embedded for histopathological analysis and processed as described^14^. Briefly, consecutive 4 µm skin sections were obtained and stained with hematoxylin-eosin, Masson’s trichrome, or Fontana-Masson (Sigma, St Louis, MO). For immunostaining, and after blocking the endogenous peroxidase activity with methanol: H_2_O_2_ (29:1), paraffin sections were incubated with 5% fetal bovine serum in PBS, then with specific primary antibodies overnight at 4 °C. Slides were washed with PBS and then incubated with biotin-conjugated secondary antibodies for 1 h. The reaction was visualized with the avidin-biotin complex (ABC) kit (Dako, Vectastain Elite; Vector Laboratories, Inc., Burlingame, CA) using diaminobenzidineas chromogenic substrate for peroxidase. Slides were stained with hematoxylin then mounted and analyzed by light microscopy (Leica DM RXA2). Quantitation of the positive p65 and β-catenin nuclei was performed by counting 100 hematoxylin-stained nuclei in 6 random areas per immunostained section. Data were expressed as percentage relative to total hematoxylin-stained nuclei (minimum of three biological replicates per experimental group).

### Immunoblotting

Frozen skin samples were used for protein isolation (whole cell extracts or cytoplasmic and nuclear fractions)^15^ after dissecting out the adipose tissue, followed by immunoblotting. Briefly, samples (30 μg/lane) were boiled in Laemmli buffer, separated on 8% SDS-PAGE and transferred to nitrocellulose membranes (Hybond ECL, GE Healthcare Bio-Sciences, Pittsburgh, PA). Then, membranes were blocked with 5% nonfat dry milk in PBS-0.1% Tween 20 and incubated with specific primary antibodies overnight at 4 °C. After washing, membranes were incubated with secondary peroxidase-conjugated antibodies, washed again and signal was detected with ECL2 (Thermo Scientific) and the ImageQuant 4000 Biomolecular Imager (GE Healthcare). Band intensities were quantitated using ImageJ software. All signals were normalized to the indicated controls (actin for whole cell extracts, or tubulin and laminA/C for cytoplasmic and nuclear extracts, respectively) with a minimum of three biological replicates. The GR activity was measured as a ratio of pGR/GR^14^.

### Antibodies

Antibodies were from Biolegend: K5 (PRB-160P), Loricrin (PRB-145P); Santa Cruz Biotechnology: p65-NF-κB (sc-372), β-catenin (sc-7199), MR (sc-11412 for ChIP), GR (sc-1004), HSD11B2 (sc-20176); Abcam: MR (ab64457; for immunohistochemistry); Cell Signalling: p-GR (Ser211; #4161), LaminA/C (#2032); and Sigma: Actin (A-2066), and Tubulin (T6199). Secondary biotin-conjugated anti-rabbit antibody (Jackson ImmunoResearch, West Grove, PA) and secondary Alexa Fluor® anti-rabbit (555, A-31572) antibody (Thermo Fisher) were used for immunostaining. Secondary peroxidase-conjugated anti-rabbit (NA934) and anti-mouse (NA931) antibodies (GE Healthcare) were used for immunoblotting.

### RNA isolation and Quantitative RT-PCR (RT-qPCR)

Total RNA was isolated from skin specimens using Trizol (Thermo Fisher) after dissecting out the adipose tissue. cDNA was generated using RevertAid H Minus Reverse Transcriptase (Thermo Fisher) and oligo dT primers (Thermo Scientific). RT-qPCR was performed in an Applied Biosystem Biosystems7500 Fast real time PCR system (Applied Biosystems, Carlsbad, CA) using specific oligonucleotides (0.3 μM each) and FastStart Universal SYBR Green Master ROX (Roche). Denaturating and annealing temperature were 95 °C and 60 °C, respectively^24^. The sequences were (5′ to 3′): TNF-A forward AGCCTCTTCTCCTTCCTGAT and reverse AAGATGATCTGACTGCCTGG, IL-6 forward GGTACATCCTCGACGGCATCT and reverse GTGCCTCTTTGCTGCTTTCAC; COX-2 forward CAGAGTTGGAAGCACTCTATGG and reverse CTGTTTTAATGAGCTCTGGATC; HSD11B1 forward TCTCCTCTCTGGCTGGGAAAG and reverse GAACCCATCCAAAGCAAACTTG, HSD11B2 forward TCAAGACAGAGTCAGTGAGAAACG and reverse GGAACTGCCCATGCAAGTG, SGK1 forward TGCTGCTGAAATAGCCAGTG and reverse CTCCTTGCAGAGTCCGAAGT, and RPLP0 forward AGATGCAGCAGATCCGCAT and reverse GTTCTTGCCCATCAGCACC. Ct values were normalized to those of RPLP0. Technical triplicates were used; and at least 3 biological replicates per experiment group were assessed to calculate the mean value ± SD.

### Cell culture and treatments

Cultured keratinocyte cell lines were previously established from adult mouse epidermis^34^. Briefly, keratinocytes were isolated from 8-wk-old female mouse dorsal skin and cultured on mitomycin C treated J2-3T3 feeders in type I collagen-coated flasks in DMEM-Ham’s F12 (3:1) medium (Thermo Fisher; Biowest, Nuaillé, France) supplemented with 1.8 × 10^−4^ mol/l adenine (Sigma), 0.35 mM calcium, 7.5% FBS Gold (Biowest), 100 U/ml penicillin/100 μg/ml streptomycin (Biowest), 2 mM glutamine (Biowest), 0.25 μg/ml amphotericin B (Biowest), 5 μg/ml insulin (Sigma), 10^−10^ M cholera toxin (Sigma) and 10 ng/ml EGF (Peprotech, Rocky Hill, NJ). Following approximately 8 passages, spontaneously immortalized lines arose.

Prior to treatments, cells were grown overnight in medium containing charcoal-stripped serum to deplete steroids, then treated with aldosterone, the GR antagonist RU486, or the MR antagonist eplerenone at indicated concentrations and times (all from Sigma). For combined treatments, cells were pre-incubated with either RU486 or eplerenone for 16 h and then treated with aldosterone for 6 h. When the two antagonists were used together with aldosterone, each reagent was used at 1 μM.

### Immunofluorescence

For immunofluorescence, cells were grown on collagen I-coated coverslips, treated with vehicle or aldosterone (100 nM, 1 h), fixed with 4% PFA and permeabilized with 0.2% Triton × 100. After blocking (PBS containing 5% donkey serum), cells were incubated with GR-specific antibody overnight at 4 °C, washed with PBS, and incubated with secondary antibody. Samples were mounted using Mowiol (Calbiochem) and DAPI (Thermo Fisher) and analyzed using a Leica DM RXA2.

### Transfection and Luciferase assays

Keratinocytes at 70–90% confluence were transfected with pGRE2EIB-Luciferase^34^, and the internal control pRL-SV40 Renilla (Promega) using Lipofectamine 2000 Reagent (Thermo Fisher). 24 h after transfection, cells were treated as indicated. Luciferase activity was measured using the dual luciferase assay system (Promega) and a Wallac Victor2 1420 multilabel counter. Firefly-luciferase levels were normalized to those of Renilla luciferase.

### Chromatin immunoprecipitation (ChIP)

ChIP experiments were performed using an established keratinocyte cell line as described^35^. Briefly, cultured keratinocytes were incubated with freshly prepared 1% formaldehyde, lysed and then sonicated using a Bioruptor (Diagenode). 3 μg of the indicated primary antibody and Dynabeads Protein A (Life Technologies) were used for immunoprecipitation. Then crosslinking reversion was performed followed by proteinase K and RNase treatment. Glycogen (Roche) was used to visualize the purified DNA pellet.

QPCR was performed to determine the relative amplification of specified genomic sites in aldosterone *vs* vehicle-treated ChIPs, which were normalized to the amplification values of respective inputs. At least 3 biological replicates were used to calculate mean ± SD. The sequences corresponding to GRE-containing Gilz regulatory regions were: forward GGAGGGAATGCAACTGGGAG and reverse CCCCTCCCTTGAATGCTGAA.

### Statistical Analysis

Experimental data were analyzed using IBM SPSS Statistics software. In all graphs, mean values ± SD are shown. When statistical analysis was performed with relative values, data were first subjected to logarithmic transformation. Prior to parametric testing, the Levene’s test was used to determine whether samples within groups had equal variance. For comparisons between two experimental groups, we used the Student’s unpaired two-tailed t-test. For comparisons among more than two experimental groups, we used the one-way ANOVA which if statistically significant was followed by a post hoc Tukey multiple comparison test. P values less than 0.05 were considered statistically significant.

### Data availability

All data generated or analyzed during this study are included in this published article (and its Supplementary Information files).

## Electronic supplementary material

Supplementary Information
