# Supplementary material for: Primary aldosteronism patients show skin alterations and abnormal activation of glucocorticoid receptor in keratinocytes
Source: Sci Rep. 2017 Nov 17;7:15806. doi: 10.1038/s41598-017-16216-5 (PMC5693903; doi:10.1038/s41598-017-16216-5)
Supplement: Supplementary file 1 — Supplementary Information [file 41598_2017_16216_MOESM1_ESM.pdf]

Primary aldosteronism patients show skin alterations and abnormal activation of glucocorticoid receptor in keratinocytes

Julia Boix[1], Judit Bigas[1], Lisa M. Sevilla[1], Maurizio Iacobone[2], Marilisa Citton[2], Francesca Torresan[2], Brasilina Caroccia[3], Gian Paolo Rossi[3], and Paloma Pérez[1, #]

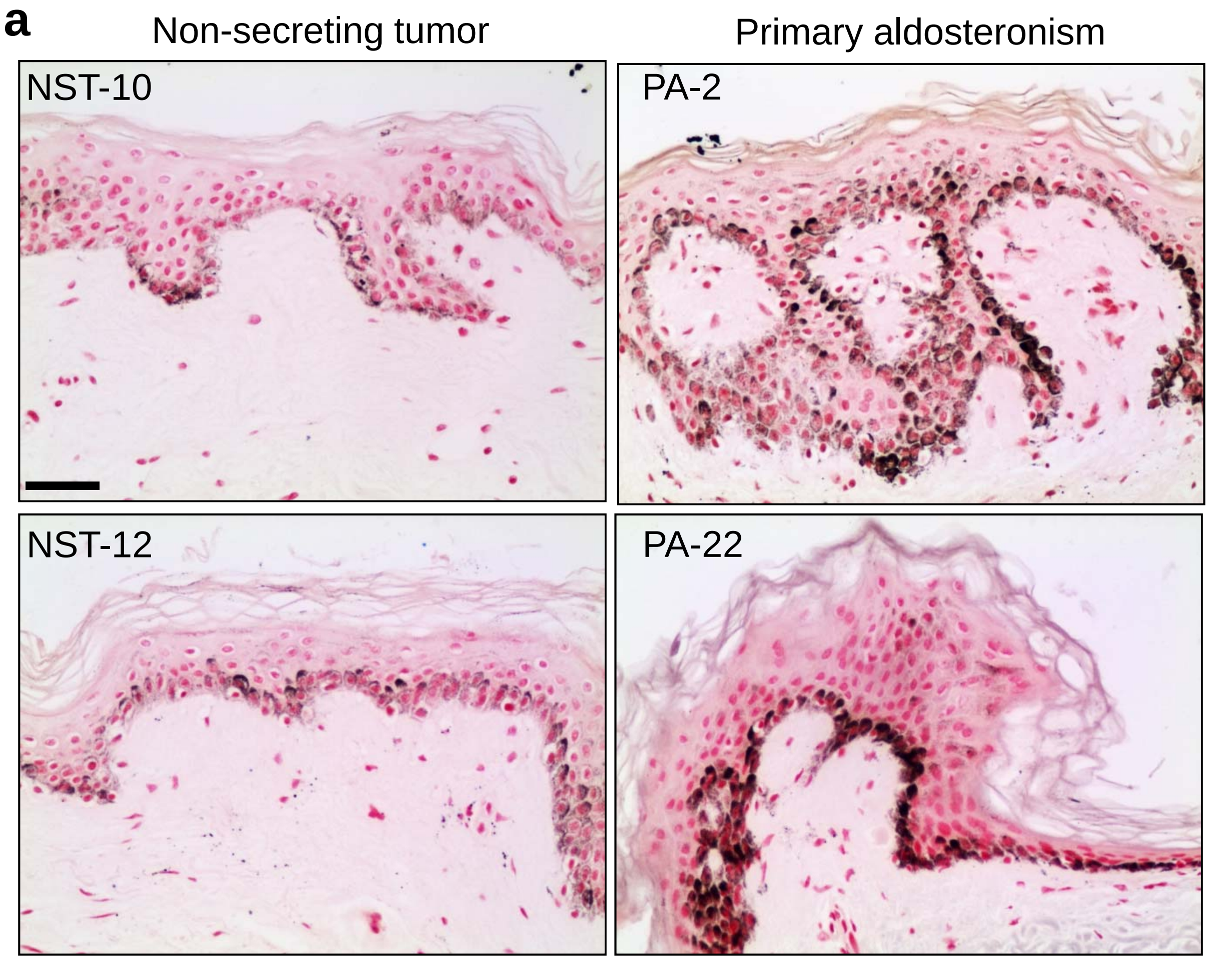

b

Sample	Patient	Score
3	NST	+/-
9	NST	+
10	NST	+
12	NST	++
16	NST	+/-
27	NST	+/-
38	NST	+
1	PA	++
2	PA	++
4	PA	+
7	PA	++
22	PA	+++
23	PA	+/-
25	PA	+++

Figure S1. Increased epidermal hyperpigmentation in the skin of PA patients
(a) Representative skin sections from control subjects (Non-secreting tumor, NST), and Primary aldosteronism (PA) patients stained with Fontana-Masson (n=7 per group). Bar: 50 μm. **(b)** Score of epidermal hyperpigmentation

Fig. 3b

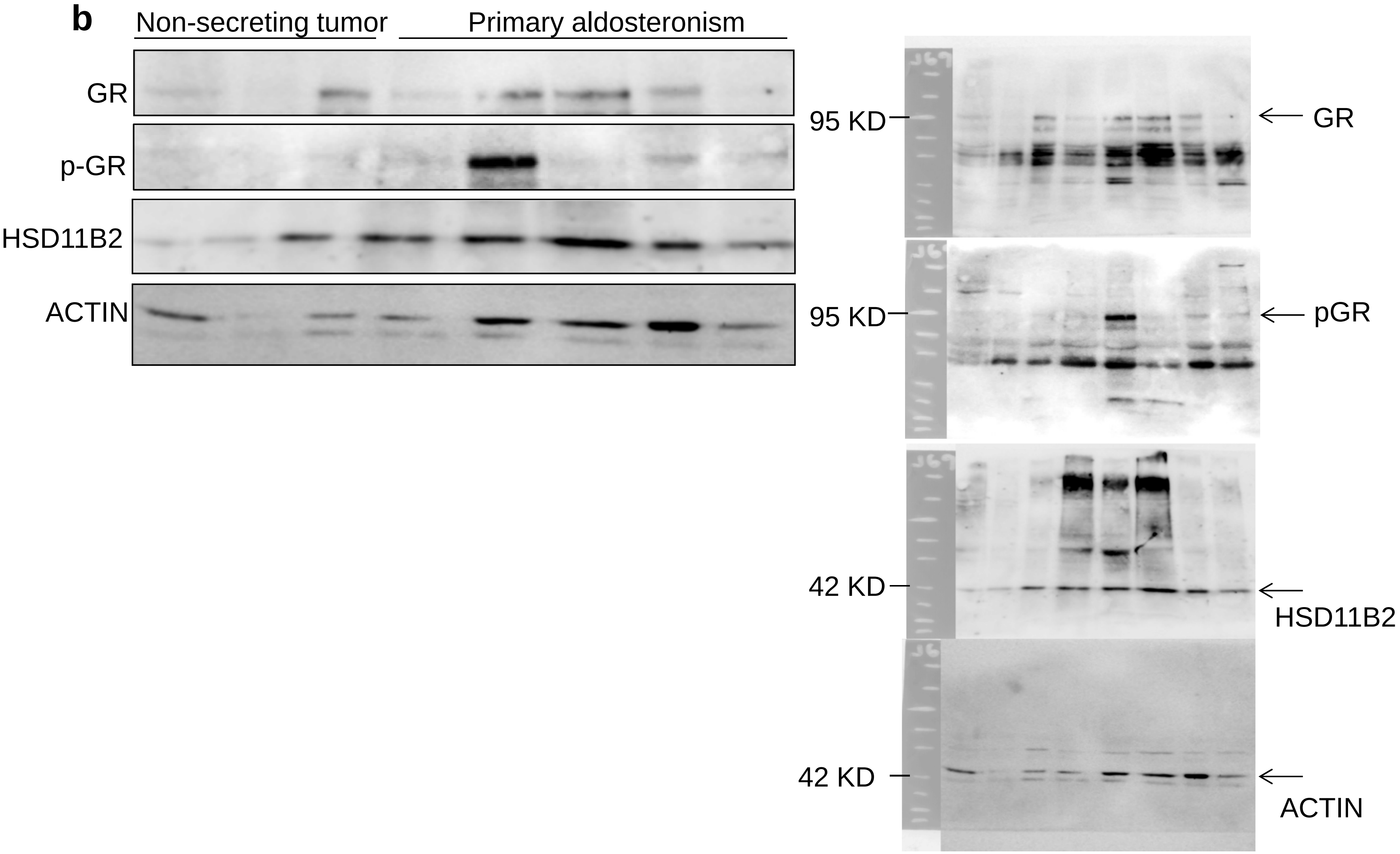

MWM: Thermo Scientific Spectra multicolor broad range protein ladder

Fig. 4a

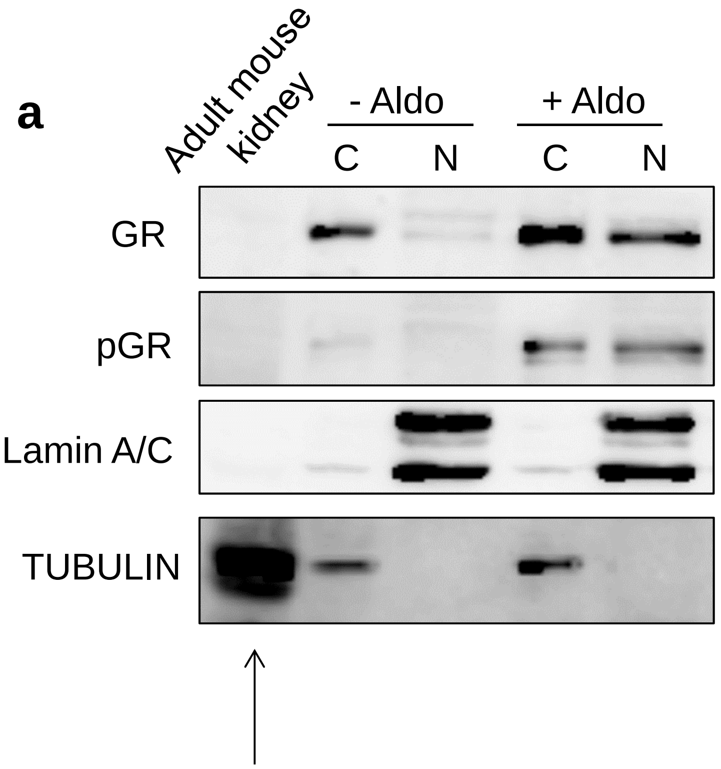

This control was not included in Fig. 4a

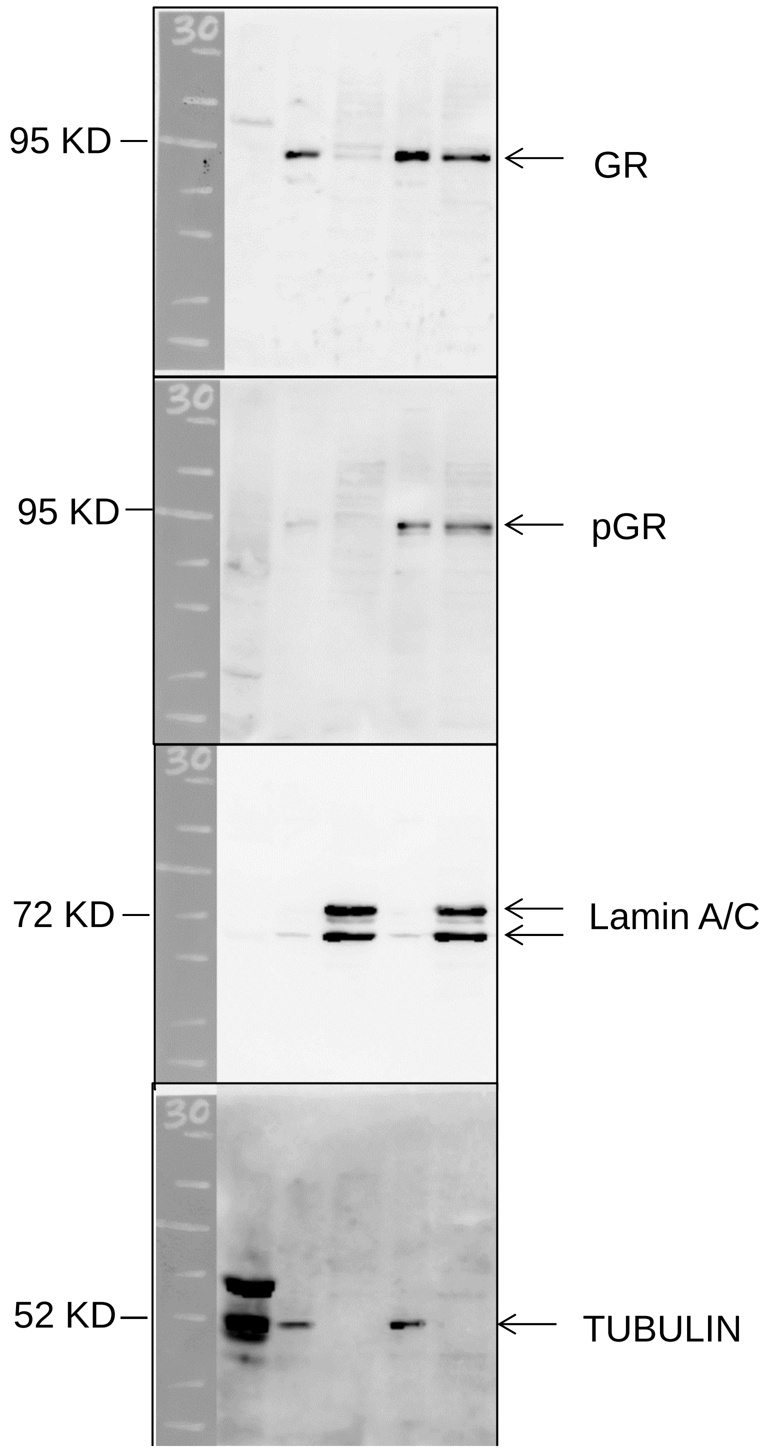

MWM: Thermo Scientific Spectra multicolor broad range protein ladder
